# TAp73β-mediated suppression of cell migration requires p57Kip2 control of actin cytoskeleton dynamics

**DOI:** 10.18632/oncotarget.833

**Published:** 2013-02-27

**Authors:** Johanna Rodhe, Edel Kavanagh, Bertrand Joseph

**Affiliations:** ^1^ Department of Oncology-Pathology, Cancer Centrum Karolinska, Karolinska Institutet, 171 76 Stockholm, Sweden.

**Keywords:** p73α, p73β, p57Kip2, actin cytoskeleton, cell migration, invasion

## Abstract

The *TP73* gene, a member of the p53 family, due to the use of different promoters and alternative splicing, is transcribed into different isoforms with contrasting attributes and which contribute to its functional diversity. Considerable efforts are made to identify the functional diversity of the p73 splicing variants during tumorigenesis.TAp73α and TAp73β isoforms have been shown to differentially regulate cell cycle progression, differentiation and apoptosis. Interestingly, a particular increase in expression of the TAp73 isoform, in favor of the α splicing variant, has been reported in multiple tumour types. Here, we report a distinctive role for TAp73β isoform in the control of cell migration and invasion. In fact, TAp73β-dependent induction of p57^Kip2^ expression accounted for inhibitory effects on the actin cytoskeleton dynamics and thereby cancer cell motility. In contrast, TAp73α is not able to induce p57^Kip2^ expression, and exhibits a positive effect on actin cytoskeleton dynamics as well as cell migration and invasion. In conclusion, the inhibitory effect on cell migration and invasion of TAp73β would qualify this distinct p73 isoform as tumor suppressor gene. In contrast, the promoting effect of TAp73α on cell motility and invasion strengthens the potential oncogenic activities of this p73 isoform.

## INTRODUCTION

The transcription factor p73 belongs to the p53 family of proteins involved in cellular responses to stress and development [1]. p73 shares a high sequence homology with the other family members, p53 and p63, and an ability to transactivate a large number of p53 target genes. p73 is a multifunctional protein, able to regulate cell cycle progression, apoptosis, and differentiation [1-3]. Similar to other family members, the *TP73* gene, due to the use of different promoters and alternative splicing, is transcribed into different isoforms with contrasting attributes and which contribute to its functional diversity [4]. There are two amino-terminally distinct types of p73 isoforms, transcriptional domain-containing (TAp73) and amino-terminal truncated (ΔNp73) isoforms directed from a downstream promoter between exons 3 and 4. ΔNp73 isoforms are thought to act in a dominant negative manner against full-length transcriptionally active TAp73 as well as wild-type p53 [5-7], although in some experimental settings ΔNp73 isoforms themselves display transcriptional activation capability [8-10]. ΔNp73 can counteract the TAp73-dependent gene expression program, either by directly binding and inhibiting transcription or by competing for DNA binding sites. Recently developed isoform specific knockout mice revealed that the depletion of TAp73 predisposes to cancer, whereas the absence of ΔNp73 impairs tumour growth in transplant assays [11, 12]. For these reasons, the relative expression level of TAp73 and ΔNp73 isoforms is considered to account for the cellular outcome of p73 gene expression. As a consequence, most studies in the field of cancer focus on analysis of changes in expression levels of TAp73 versus ΔNp73 forms of p73. However, surprisingly, consistently higher expression of TAp73 isoforms is found in the vast majority of cancer cell lines [13]. Furthermore, the overexpression of the ΔNp73 isoform α in human colon carcinoma cells does not induce a more aggressive phenotype *in vivo* or affect the response of these cells to anticancer agents [14].

One should keep in mind that the p73 transcripts undergo alternative splicing, which generates different proteins which share the same amino-terminal and central DNA binding domain, but differ in a variety of carboxy-terminal portions (termed α to ζ) [4]. However, p73α and p73β are the two main p73 full-length isoforms expressed in human cells. Interestingly, these two p73 isoforms have been shown to differentially regulate cell cycle progression [15] and differentiation [16]. In addition, in various cellular contexts, the p73β isoform appears a better cell death promoting factor as compared to the p73α isoform. It has also been reported that p73α can hold anti-apoptotic function in small cell lung carcinoma cells [17-19] and ovarian carcinoma [20]. Collectively these studies revealed that full-length p73α and p73β can differentially affect various hallmarks of cancer cells [21]. Cell migration is a key aspect of many normal and abnormal biological processes, including invasion and metastasis of tumor cells [22]. It is generally accepted that the driving force for the cell movement is provided by the dynamic reorganization of the actin cytoskeleton. Overexpression of p73α has previously been shown to promote cell migration [23], whereas the effect of p73β on cell migration is as yet unknown. It is of importance to define the distinct effect of the individual full-length p73 isoforms on this biological process, in order to understand the contribution of each isoform to oncogenesis.

## RESULTS

### p73β, but not p73α isoform expression inhibits cell migration

**Figure 1:**
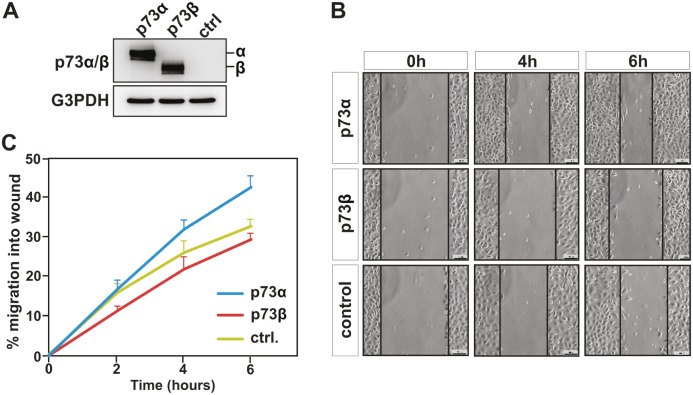
p73β, but not p73α expression, inhibits cell migration Human cervical cancer HeLa cells were transfected with expression vector encoding for p73α or p73β, and compared with cells that were transfected with the backbone pcDNA3 expression vector. Protein expression was confirmed by immunoblotting against p73α/β, using G3PDH as loading control (A). Cell motility was determined by wound-healing assay measuring cell migration into the wound (B). Data shows mean values of three independent experiments +SEM (C). At 6 hours, statistical analysis of p73α versus p73β indicates a p-value ** <0.01.

Overexpression of p73α has previously been reported to promote cell migration of colon carcinoma HCT116 and non-small cell lung carcinoma H1299 cells [23]. The consequence of p73β expression on cell migration is however unexplored. Despite the fact that p73α and p73β share common features they also have distinct functions [4, 25]. In fact, these two isoforms have specific transcriptional target genes and interacting partners, which may lead to different regulation of cellular processes. We therefore decided to examine whether p73β can also influence the migration ability of the cells. Confluent human cervical carcinoma HeLa cell monolayers were subjected to a wound-healing assay to monitor cell motility. Transient transfection of expression vector encoding p73α or p73β in HeLa cells (Figure 1A), was performed 24 h before wounding. Confluent cell cultures were scraped with a pipet tip to create a cell-free wound and images were captured at the beginning and at regular 2 hours intervals during cell migration to close the wound. A decrease in wound healing (cell motility) activity was clearly seen in cells expressing p73β already after 2 h as compared to control cells (Figure 1B-C). Quite the opposite, p73α expressing HeLa cells exhibited a robust increase in velocity as compared to both control and p73β expressing cells. At 6 h, p73α expressing cells reduced the wound area by 43+/−3 %, and control cell by 33+/−2 %, while the p73β expressing cells exposed region was measured to be reduced 29+/−2 % of the initial distance between the edges of wound area (Figure 1C). Thus, these data establish that p73β expressing cells have a reduction in cell migration as measured in this classical wound-healing assay.

### p73β expression reduces cell invasion

**Figure 2:**
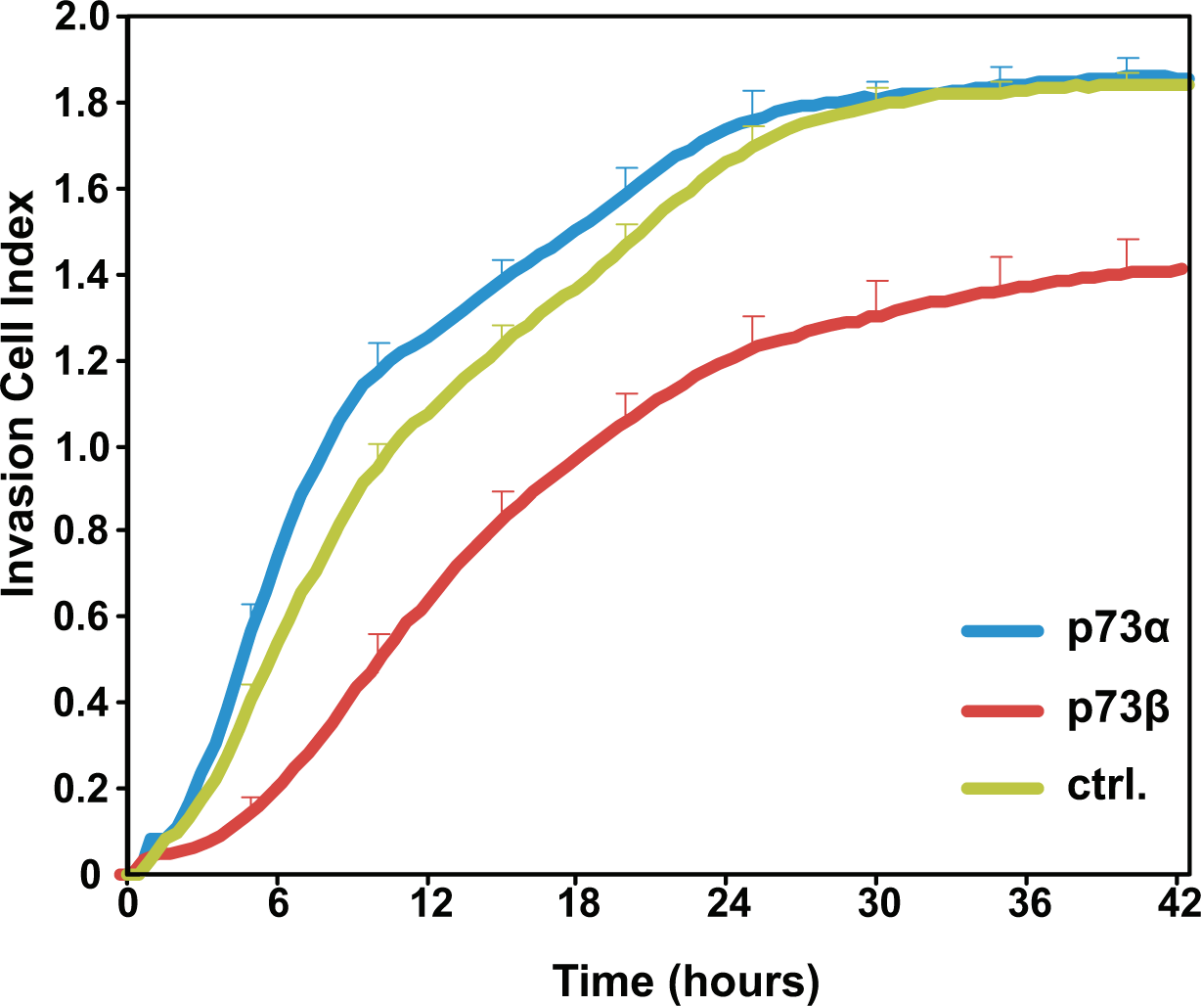
p73β expression reduces cell invasive capacity The invasive ability of p73α- and p73β-expressing HeLa cells as compared to control pcDNA3 transfected HeLa cells were determined in real-time during 42 h, using Matrigel-coated transwell chamber and the xCELLigence RTCA DP analyzer. Cell Index represents the degree of cell invasion into the reconstituted basement membrane matrix Matrigel. Data shows representative invasion curves for each condition performed in triplicate +SD, out of three independent experiments. At 24 hours, statistical analysis of p73α versus p73β indicates a p-value * <0.05.

Invasion of cancer cells through the extracellular matrix is a critical step in tumor metastasis. Cell invasion is related to, and encompasses, cell migration, however cell invasion cannot be restricted to this process. Indeed, invasive cells move through the extracellular matrix into neighboring tissues in a process that involves extracellular matrix degradation and proteolysis. Thus, we went on and investigated whether the observed decrease in cell migration observed in p73β expressing cells was associated with a reduced invasion ability of these cells. HeLa cells were transiently transfected with expression vectors for p73α or p73β. A modified matrigel-coated Boyden chambers system was used to quantify in real time the invasive ability of HeLa cells expressing various p73 isoforms. Cell invasion of serum starved cells, migrating through a matrigel towards 10 % FBS, was monitored over a 42 hour period of time. Remarkably, p73β expression leads to a robust reduction in the ability of HeLa cell to invade the reconstituted basement membrane (Matrigel) as compared to mock transfected cells. The inhibitory effect on cell invasion was already visible after 3 hours and was sustained up to 42 hours (Figure 2). In contrast, p73α expressing cells exhibited a modest but significant increase in cell invasion capability as compared to control cells. These data provide compelling evidence that the expression of selective p73 isoforms in cancer cells can lead to contrasting effects on the abilities of cells to migrate and invade a reconstituted basement membrane.

### Actin cytoskeleton dynamics are impaired in p73β expressing cells

**Figure 3:**
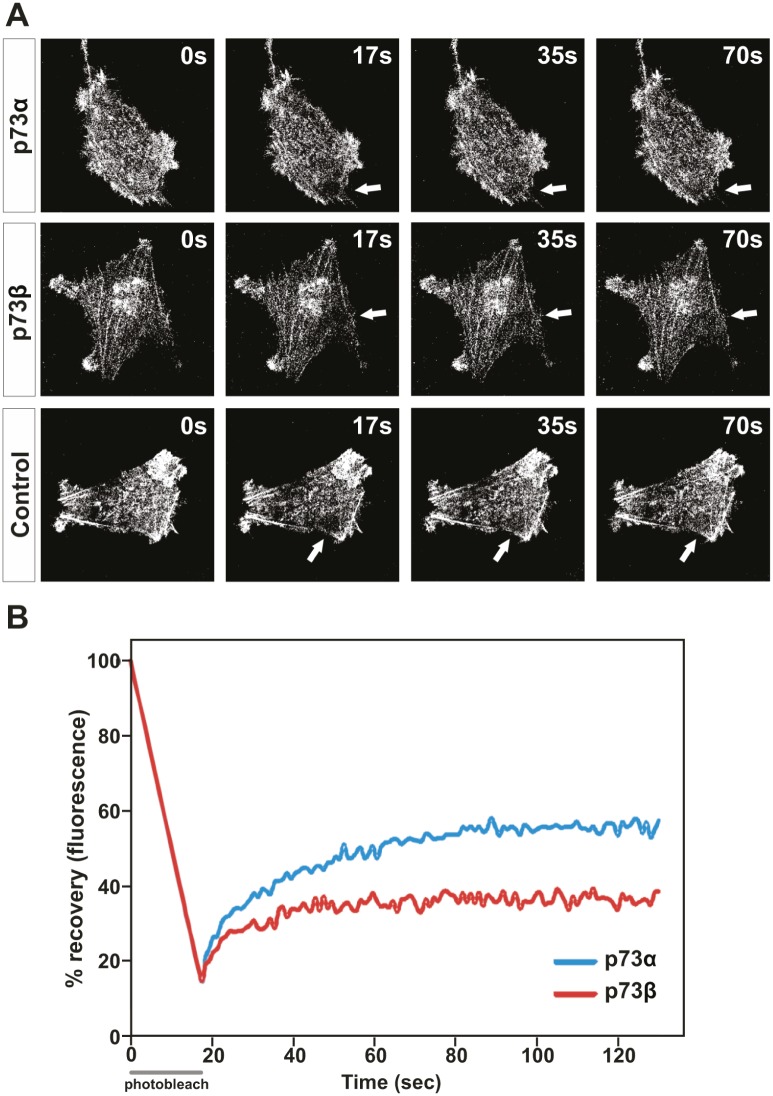
Actin cytoskeleton dynamics are impaired in p73β-expressing cells HeLa cells were transfected with expression vector encoding for p73α or p73β, and compared with control cells transfected with the corresponding empty vector. The mobile actin fraction in p73α-, or p73β-expressing HeLa cells was measured by FRAP analysis of cells co-transfected with actin-GFP to visualize the actin cytoskeleton. Values represent the % fluorescence recovery over time of actin-GFP after bleaching. Arrows indicate the photobleached area (A). For every FRAP experiment 3-7 cells per condition were used. Average of three independent experiments is presented in the figure (B).

Although cell migration cannot be attributed to actin polymerization alone, the redistribution of actin fibers and the formation of pseudopodia are important events in cell locomotion. The actin cytoskeleton is believed to provide both the protrusive and contractile forces required for cell migration, via a combination of actin polymerization and depolymerization. Actin dynamics and turnover rates have been implicated in many aspects of cell function. This dynamic relationship can be investigated using fluorescence recovery after photo bleaching (FRAP) of fluorescently labeled actin. We proceeded by looking at the actin turnover kinetics in p73α expressing *vs.* p73β expressing cells. HeLa cells were co-transfected with a GFP tagged actin expression vector together with a p73 expression vector. In these experiments, a delimited circular region that occupied 7,43 μm of diameter in a GFP-actin expressing HeLa cells was photobleached by an intense laser radiation and the exchange between the bleached and the unbleached population of GFP-actin was then monitored. In each case, the recovery of fluorescence at the bleached area was monitored for at least 150 s after laser irradiation (Figure 3A). In p73α expressing cells, the maximum fluorescence recovery at the bleached area was reached within 80 s. In contrast, the exchange between the bleached and the unbleached region in p73β expressing cells was significantly slower and was not able to attain the levels of the p73α expressing cells during the monitoring period (Figure 3B). Performing FRAP analysis on cells expressing GFP-tagged proteins can reveal information regarding the turnover kinetics of the labeled population as well as the fraction of the population that participates in turnover (mobile fraction, α). In fact, selective p73β expression in HeLa cells resulted in a fall of the actin mobile fraction from α=0.41 in p73α expressing cells to α=0.20 in p73β expressing cells. These data suggest that p73β expressing cells, which have more actin engaged in the formation of stress fibers, have a lower pool of free actin available. Cells continuously control the growth and shrinkage of actin filament networks in order to perform tasks crucial for their survival such as cell motion. Thus, the diminished actin dynamics in cells expressing p73β could have implications for this cellular function.

### p73β is dependent on p57^Kip2^ induction to regulate actin cytoskeleton dynamics

**Figure 4:**
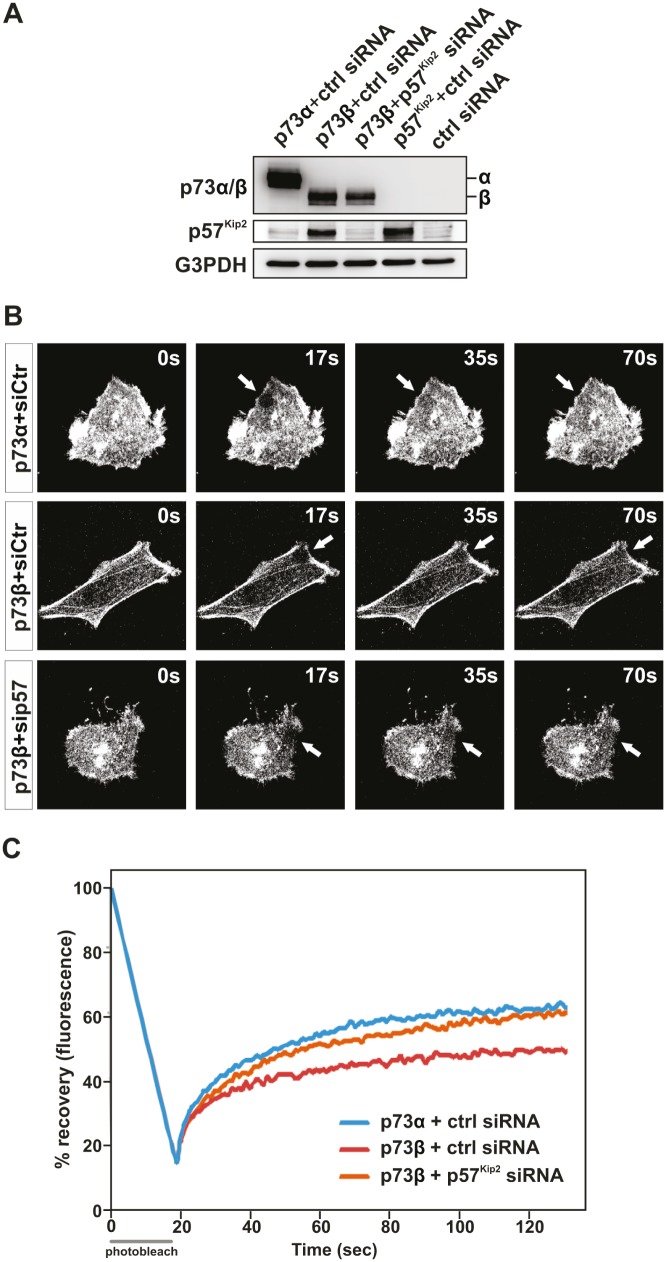
p73β regulation of the actin cytoskeleton dynamics is dependent on induction of p57^Kip2^ HeLa cells were co-transfected with the expressing vector encoding for p73β, together with a p57^Kip2^ siRNA or a scrambled sequence siRNA. p57^kip2^ expression and knockdown was confirmed by immunoblotting against p57, using G3PDH as a loading control. HeLa cells co-transfected with the expressing vector encoding for p73α, p57^Kip2^ or the backbone expression vector together with the scrambled sequence siRNA were used as controls. (A). FRAP assay on GFP-actin-expressing cells was performed to evaluate actin cytoskeleton dynamincs. Bleached area of actin-GFP is indicated by an arrow (B). The actin turnover is measured as % recovery of the actin-GFP signal during 150 s (C). For every FRAP experiment 4-7 cells per condition were used. Average of three independent experiments is presented in the figure (C).

In the present study, we reveal that p73β and p73α exhibit contrasting effects on the actin cytoskeleton dynamics. Accumulating evidences also reveal the existence of an isoform-specific regulation of target genes. Indeed, p73β and p73α, acting as transcription factors, demonstrate selectivity on the regulation of gene expression [19, 24, 26-30]. Of interest for this study, p73β has been shown to be a potent transactivator of the *p57^Kip2^* gene [24, 26-28, 30], whereas p73α was found to be inactive in this aspect (Figure 4). Originally described as a cyclin dependent kinase inhibitor, p57^KIP2^ has since been shown to influence other cellular processes beyond cell cycle regulation, including cell death and cell migration [24, 30-34]. We previously reported that p57^Kip2^ inhibits cancer cell migration through stabilization of the actin cytoskeleton in a LIM-Kinase-1 dependent manner [24, 34]. p73β-induced p57^Kip2^ expression could provide one possible explanation to how p73β can regulate the actin cytoskeleton dynamic and thereby cell mobility and invasion capability. In order to substantiate the involvement of p57^Kip2^ in the control of actin cytoskeleton dynamics by p73, the effect of silencing p57^Kip2^ with small interference RNA (siRNA) was investigated. For this purpose, HeLa cells were co-transfected with p57^Kip2^, p73β, p73α or empty expression vector together with a siRNA designed to interfere with the expression of human p57^Kip2^ or a control siRNA (Figure 4A). Control siRNA did not affect p57^Kip2^ expression level. Immunoblotting analysis revealed that siRNA against p57^Kip2^ significantly reduced the level of p57^Kip2^ protein in p73β expressing cells. Furthermore, siRNA-mediated knock-down of p57^Kip2^ significantly reduced the formation of actin stress fibers associated with p73β expression in HeLa cells, suggesting that indeed p73β induction of p57^Kip2^ expression accounts for its effect on the actin cytoskeleton (Figure 4B). To strengthen this observation, FRAP analysis was performed. HeLa cells were co-transfected with a GFP tagged actin expression vector together with expression vectors encoding p73α or p73β, and siRNA targeting p57^Kip2^ or a control siRNA. Remarkably, silencing of p57^Kip2^ in p73β expressing HeLa cells abrogated the inhibitory effect of p73β expression on the actin dynamics and turnover rates (Figure 4C). In fact, suppression of p57^Kip2^ expression in p73β expressing HeLa cells restored the actin mobile fraction to levels comparable to p73α expressing cells. Silencing of p57^Kip2^ also enhanced the kinetic of recovery in the p73β expressing HeLa cells. Altogether, these data provide compelling evidence that the inhibitory effect of p73β on actin cytoskeleton dynamics requires p57^Kip2^ induction and its regulatory effect on the actin cytoskeleton.

## DISCUSSION

The individual functions of the different full length (*i.e.* TA domain containing) p73 carboxy-terminal isoforms are emerging from the compelling evidences of numerous recent studies, which highlight that these carboxy-terminal isoforms differ in regard to relative efficiency in transactivation of promoters of target genes, post-translational modifications, and interaction partners. For example, we have shown that p73α can regulate expression of the *HSP72* gene, an activity not shared by p73β [19]. On the other hand, p73β has been shown to be a potent transactivator of the *Aquaporin 3* [29], caspase-3 [35] *Rb1*, *P21^waf1/Cip1^* [36] and *P57^Kip2^* [24, 26, 27] genes, whereas p73α is inefficient in this aspect. The final outcome of these variations among the TAp73 carboxy-terminal isoforms are ultimately displayed as different potencies in the induction of apoptosis, cell cycle arrest, and/or differentiation [37-39]. Of note, p57^Kip2^ can promote drug-induced cell death [30, 33, 34], providing one possible explanation for why TAp73β, in many cases, is a more potent inducer of apoptosis than TAp73α [40].

In this study, we uncover a completely novel and distinctive role for p73β isoform in the control of cell migration and invasion. We demonstrate that the p73β-dependent induction of p57^Kip2^ expression accounts for a negative effect on actin cytoskeleton dynamics and thereby cancer cell motility. In contrast, p73α is unable to induce p57^Kip2^ expression, exhibits a positive effect on actin cytoskeleton dynamics as well as cell migration and invasion. Overall, the death-promoting effect and the inhibitory effect on cell migration and invasion of p73β would categorize this distinct TAp73 isoform as tumor suppressor gene. Quite the opposite, the reduced or even reported inhibitory effect of p73α on apoptosis together with its promoting effect on cell motility and invasion would argue for oncogenic activities for this particular TAp73 isoform [23].

Whether these functional differences between the TAp73 isoforms might contribute to tumorigenesis is of interest. As mentioned earlier, altered expression of the p73 gene has been reported in hematologic and solid tumors. Remarkably and of particular interest for the current study, increased TAp73alpha expression levels have been noticed in certain cancers like medulloblastoma [41], B-cell chronic lymphocytic leukaemia [7], cervical cancer [42], ovarian carcinomas [43], gastric adenocarcinoma [44], bladder cancer [45], prostate cancer [46] and thyroid cancer [47]. In addition, consistently higher expression of endogenous TAp73α isoforms is found in the vast majority of cancer cell lines [13]. Furthermore, a shift in the expression of p73 isoform mRNA levels from exon 13 lacking (*i.e.* p73β) to exon 13 containing copies (i.e. p73α) has also been reported in prostate cancer cases as compare to normal prostate [46]. Some of the functional differences between TAp73 isoforms, e.g. TAp73α and TAp73β, are likely to be explained by the specific interactions of TAp73α’s unique domains, *i.e.* sterile α motif (SAM) domain and extreme carboxy-terminus, with other proteins. Finally, the *P73* gene, depending on the isoforms expressed, appears to have a Jekyll and Hyde behaviour in cancer cells. Thus, TAp73α and TAp73β should be defined as individual transcription factors and further efforts should be placed on the understanding of their unique and sometimes rather contrasting behaviour to make the most use of drugs and cancer treatments.

## MATERIAL AND METHODS

### Cell culture

Human cervical carcinoma HeLa cells (ATCC CCL-2) were maintained at 37 °C, 5 % CO2 in Minimum Essential Medium (MEM) (Gibco) supplemented with 10 % heat-inactivated fetal bovine serum, 1 mM sodium pyruvate, 1 % non-essential amino acids, 2 mM L-glutamine, penicillin (100 U/ml) and streptomycin (100 μg/ml).

### Transfection

Plasmids encoding HA-p73α (pcDNA_3_-HA-p73α) and HA-p73β (pcDNA_3_-HA-p73β) were gifts from Dr. G. Melino (University of Tor Vergata, Italy) and have been described [3]. pcDNA_3_-HA-p57^Kip2^ was a gift of Dr. Y. Xiong (University of North Carolina, USA). GFP-actin was gift of Dr. P. Hotulainen (University of Helsinki, Finland) [24]. EGFP and pcDNA_3.1_ plasmids used as controls were from Clontech and Invitrogen respectively. ON-TARGET plus SMARTpools siRNA against human p57^Kip2^ and control ON-TARGET plus Non-targeting siRNA #1 were purchased from Dharmacon. Twenty-four hours after seeding into culture dishes with fresh medium, cells were transfected using Lipofectamine PLUS (Invitrogen) according to the manufacturer’s protocol. Transfection and siRNA knockdown efficiencies were both confirmed by immunoblotting.

### Protein extracts and immunoblotting

Cells were harvested and lysed by sonication in NP40 lysis buffer containing protease inhibitors. Protein extracts were heated to 95 °C for 5 min in Laemmli buffer. Samples were resolved on 12 % SDS-polyacrylamide gels and blotted onto nitrocellulose membranes. Membranes were incubated with a blocking buffer (5 % milk powder, 0,1 % Tween in PBS) for 1 hour and incubated with anti-p73α/β (Ab4; Neomarkers), anti-p73α/β (Ab2; Neomarkers) or anti-p57 (C-20; Santa Cruz Biotechnology) antibodies overnight at 4 °C, followed by incubation with the appropriate horseradish peroxidase secondary antibody (Pierce) for 1 h at room temperature. Immunoblot with anti-G3PDH antibody (Trevigen) was used for standardization of protein loading. Bands were visualized by enhanced chemiluminescence (ECL Plus) following the manufacturer’s instructions (Thermo scientific) using the digital imaging system Image Quant LAS 4000 (GE Healthcare).

### Measurements of wound healing (cell motility) activities

HeLa cells were grown to 75 % confluence in 60-mm dishes at time for transfection. Twenty four hours post-transfection, when cell culture had reached 95 % confluence, one site in each dish was scraped with a 200 μl plastic pipette tip to create a “wound” cleared of cells. The medium was removed and was replaced with fresh medium. The progress of cells moving into the wound area was photographed at 0h, 2h, 4h and 6h using Olympus CK2 inverted microscope with Olympus Delta-Pix Inventio 3S at 10x magnification. For each scrape, three measurements across the “wound” were taken at each time point, and three independent experiments were performed.

### Fluorescence recovery after photobleaching (FRAP)

FRAP was applied to measure the actin treadmilling rates in HeLa cells as previously described [24]. HeLa cells were grown on 60 mm coverslips to 50 % confluence and thereafter co-transfected with GFP-actin and p73α, p73β or pcDNA_3.1_ empty vector subsequently for 24 h, and placed on glass chamber slide. Chambers were then placed in the POC-chamber/CTI Controller/Heating insert P system for live cell imaging. After bleaching of the region of interest (ROI), a circular area of 7,43 μm in diameter, with 100 % intensity of Argon/2 laser 488 nm (current 4.7 Amp), the time course fluorescence recovery in the bleached ROI was monitored with 20 ms interval during 150 s. Samples were analyzed under Zeiss 510 Meta confocal laser scanning microscopy equipped with an inverted Zeiss Axiovert 200m microscope with a 63x oil immersion objective.

### Real-time cell invasion assay

Real-time cell invasion was monitored on the xCELLigence RTCA DP Instrument using a 16-well modified Boyden chamber CIM-Plate 16 composed of an upper chamber and a lower chamber. Briefly, 24 hours post transfection, cells were serum starved for 4 hours and detached by Versene before loaded on the upper chamber of a transwell CIM-plate 16 at 45.000 cells/well. The 8 μm pore wells were coated with basement membrane matrix Matrigel diluted 1:30 (BD Biosciences), and the cells were migrating towards 10 % FBS in the lower chamber. Invasion was measured every 30 min for 42 hours by changes in the measured Cell Index values as analyzed by RTCA software 1.2.1.

### Statistical analysis

Statistical analyses were performed using two-tailed, paired students t-test, where ***p-value <0.001, **p-value <0.01 and *p-value <0.05.
